# What Electrochemical Biosensors Can Do for Forensic Science? Unique Features and Applications

**DOI:** 10.3390/bios9040127

**Published:** 2019-10-29

**Authors:** Paloma Yáñez-Sedeño, Lourdes Agüí, Susana Campuzano, José Manuel Pingarrón

**Affiliations:** Departamento de Química Analítica, Facultad de CC. Químicas, Universidad Complutense de Madrid, E-28040 Madrid, Spain; malagui@quim.ucm.es (L.A.); susanacr@quim.ucm.es (S.C.); pingarro@quim.ucm.es (J.M.P.)

**Keywords:** electrochemical biosensors, forensic analysis, poisons, drugs, toxins, explosives, chemical and biological weapons

## Abstract

This article critically discusses the latest advances in the use of voltammetric, amperometric, potentiometric, and impedimetric biosensors for forensic analysis. Highlighted examples that show the advantages of these tools to develop methods capable of detecting very small concentrations of analytes and provide selective determinations through analytical responses, without significant interferences from other components of the samples, are presented and discussed, thus stressing the great versatility and utility of electrochemical biosensors in this growing research field. To illustrate this, the determination of substances with forensic relevance by using electrochemical biosensors reported in the last five years (2015–2019) are reviewed. The different configurations of enzyme or affinity biosensors used to solve analytical problems related to forensic practice, with special attention to applications in complex samples, are considered. Main prospects, challenges to focus, such as the fabrication of devices for rapid analysis of target analytes directly on-site at the crime scene, or their widespread use and successful applications to complex samples of interest in forensic analysis, and future efforts, are also briefly discussed.

## 1. Introduction

A broad spectrum of sciences is used in forensic investigations, with the objective of providing answers to questions of interest related to a crime or a civil action. Among these, forensic analysis, which currently constitutes a significant branch of modern analytical chemistry, makes use of different techniques, including liquid and gas chromatography [1,2], spectroscopy [3], and electrochemistry [4] to obtain information that implies many important social and legal consequences. In this field, biosensors have become ideal tools not only for rapid initial screening but also for sensitive determination of suspicious agents due to the biosensors’ great advantages of specificity, rapidity, and little sample manipulation [5]. A variety of recognition elements such as enzymes, antibodies, and sequences of nucleic acids, as well as different transduction techniques, mostly optical or electrochemical, to convert the bio-recognition event into a measurable signal, are available. 

Furthermore, biosensing detection is particularly suitable for the quantitative analysis of chemical or biochemical species, including genetic material, blood, saliva, urine, sweat, or semen, which are common samples in forensic analysis. At present, electrochemical biosensors have been reported for the detection and quantification of most target compounds of interest in forensic analysis. However, despite their advantages, the real applications of biosensors in this field are still scarce [6], and, in several occasions, the biosensors have not been validated for the analysis of complex samples.

Electrochemical biosensors exhibit advantageous features inherent to the electrochemical detection, such as high sensitivity, great precision and accuracy, easy handling, low cost, minimal sample requirement, simple integration into portable platforms, low power consumption, and multiplexing capabilities [7], which make them extremely attractive in forensic analysis. In addition, coupling of electrochemical transduction with the use of nanomaterials and magnetic microcarriers leads to significant improvements in the conductivity of the sensor and the ability for immobilization of biomolecules [8]. This article reviews the role of electrochemical biosensors in forensic analysis during the last five years. The applications to forensic toxicological analysis are classified by the nature of the target analytes, and the developed methods for the detection of chemical explosives, gunshot residues, fire accelerants, warfare agents, and biological weapons are reviewed and critically discussed. Informative tables provide relevant characteristics of the highlighted methodologies. 

## 2. Electrochemical Biosensors Applied to Toxicological Forensic Analysis

### 2.1. Inorganic Poisons: Arsenic and Cyanide

Arsenic is one of the most abundant elements and is present in various minerals and in combination with metals of high industrial usefulness. However, inorganic arsenic, especially in the form of As(III) is highly poisonous and a toxic carcinogen [9,10]. A large number of people across the world is currently affected by arsenic contamination and at risk of arsenic poisoning due to exposure to polluted drinking water. The World Health Organization (WHO) and the Environmental Protection Agency (EPA) set the maximal standards of As(III) level in drinking water at 10 ppb in 2006 [11]. This low concentration has prompted the development of sensitive analytical methods for As(III) determination. As Table 1 summarizes, among the methods based on the use of biosensors, aptasensors stand out in recent years [12,13]. Furthermore, similarly to other approaches for the detection of toxic chemicals, electrodes modified with nanomaterials have been utilized for the specific and selective recognition of arsenic by immobilizing a specific biorecognition element [14]. Electroanalytical inorganic arsenic speciation, including the use of biosensors, was reviewed by Antonova and Zakharova [15]. Unfortunately, the reported biosensors are mostly applied to environmental samples, and no application to biological samples has been found in recent years.

**Figure 1:**
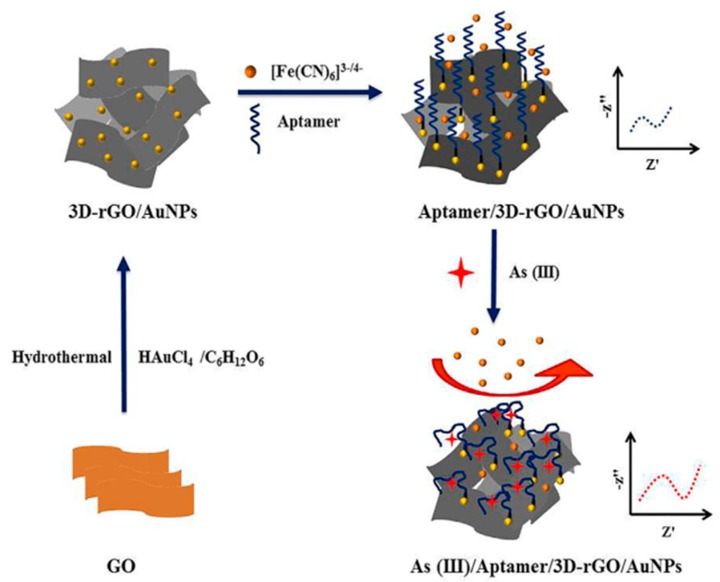
Scheme of the preparation and functioning of an As(III)/Aptamer/3D-RGO/AuNPs aptasensor. Reprinted from [19], with permission.

As it is known, aptamers are single-stranded DNA or RNA oligonucleotides synthesized by a combinatorial selection process called SELEX (Systematic Evolution of Ligands by EXponential enrichment) [16]. The development of aptasensors for forensic analysis was revised by Gooch et al. [17]. Important practical advantages of these molecules are high stability and easy modification. A recent aptamer-based electrochemical biosensor for the determination of As(III) is the one reported by Baghbaderani and Noorbakhsh [18], involving the use of a chitosan/Nafion-modified glassy carbon electrode as scaffold for the immobilization through glutaraldehyde cross-linking of a capture DNA probe complementary to the arsenic-specific aptamer. A nanocomposite using carboxylated carbon nanotubes was employed for electrochemical impedance spectroscopy (EIS) signal amplification after hybridization with the specific aptamer, providing a detection limit of 74 pM. Another recently reported electrochemical aptasensor for the determination of As(III) involves three-dimensional reduced graphene oxide (3D-rGO) modified with gold nanoparticles (3D-rGO/AuNPs) for the immobilization of a thiolated aptamer via Au–S covalent binding (Figure 1). Porous 3D-rGO/AuNPs with large active surface area were prepared by hydrothermal treatment of GO in the presence of HAuCl_4_ and glucose. In the presence of As(III), EIS signals of the aptamer/3D-rGO/AuNPs/GCE increased due to the hindered electron transfer after As(III) binding to the immobilized aptamer. A calibration plot was constructed, showing linearity between the variations of charge transfer resistance (ΔR_CT_) and the logarithm value of As(III) concentration over the 3.8 × 10^−7^–3.0 × 10^−4^ ng·mL^−1^ range. The limit of detection (LOD) value was 1.4 × 10^−7^ ng·mL^−1^ [19]. 

An electrochemical aptasensor involving As(III)-specific aptamer for recognition and signal amplification mediated by hybridization chain reaction (HCR) and RecJf exonuclease catalyzed reaction was also reported. DNA assembly was made on a gold electrode surface, provoking a big charge in R_CT_. The As(III) triggers the dissociation between the aptamer sequence and DNA. The release of HCR product significantly decreased R_CT_, which could be further enhanced by RecJf exonuclease catalyzed digestion. The electrochemical response originated from the variation of R_CT_ across the modified electrode. Ultrasensitive detection of As(III) was achieved with an LOD as low as 0.02 parts per billion (ppb), 500 times below the content limit of 10 μg·L^−1^ (10 ppb) recommended by WHO for drinking water [20].

Regarding cyanide, it is known to be an extremely poisonous substance due to its high affinity toward the iron ions and the suppression of oxygen transport [21]. Accidental cyanide release in wastewater or rivers may lead rapidly to serious contamination of groundwater and even drinking water. However, due to its excellent properties, cyanide is widely used in a variety of applications. A representative example of methods for cyanide determination, involving electrochemical biosensors, is an amperometric strategy based on cyanide’s inhibitory effect on the activity of catalase. The enzyme was immobilized onto a platinum electrode, modified by aniline polymerization, to obtain the conductive PANI polymer, which acts as a redox mediator in the H_2_O_2_ detection and blocks the interferences from reducing agents present in real samples [22]. The amperometric responses correlated linearly with the cyanide concentration between 16.3 × 10^−3^ and 0.65 mg·L^−1^. In addition, a potentiometric biosensor for cyanide was developed, taking advantage of the dehydratase activity of whole *Flavobacterium indicum* cells, which were coupled to an ammonium ion selective electrode. By using the agar immobilized whole cell as the biocomponent, the potentiometric biosensor detected a low cyanide concentration of 0.06 ppm, with a response time of 2 min [23].

### 2.2. Organic Toxics: Alcohol

Alcohol is one of the most common poisonous compounds consumed by human beings that is closely related to health damages and traffic accidents [27]. Over the years, the need for a fast and reliable measurement of ethanol in biological samples has become of high importance in clinical and forensic medicine [28]. Much advancement in the improvement of biosensors for this purpose was witnessed in recent decades, until reaching the current wearable biosensors [29]. Currently, electrochemical biosensors dominate the field which harnesses the synergistic action of specific enzymes with efficient catalytic properties and nanomaterials for the analysis of ethanol. Recent insights into amperometric enzyme biosensors for alcohol quantification related to novel electrode materials and different immobilization strategies were reviewed by Hooda et al. [30]. 

The two major enzymes involved in catalytic reaction of ethanol for electrochemical measurements are alcohol oxidase (AOx) and alcohol dehydrogenase (ADH). Among the recent strategies, it is worth highlighting the application of new nanomaterials that improve the analytical characteristics of the detection. An example is the use of single-walled carbon nanotubes (SWCNTs), covalently functionalized with polytyrosine for the construction of an ethanol biosensor through immobilization of ADH via Nafion entrapment and amperometric detection in the presence of NAD^+^. The electrode material exhibited electrocatalytic activity toward NADH oxidation due to the effect of the quinones generated from the primary oxidation of tyrosine. Therefore, a potential value as low as 0.2 V vs. Ag/AgCl was applied for the amperometric detection, reaching an LOD of 0.67 mM [31]. In a similar configuration, Bilgi and Ayranci [32] prepared a SPCE modified with multiwalled carbon nanotubes (MWCNTs), gold nanoparticles (AuNPs), and polyneutral red (PNR) film for the construction of a disposable ethanol biosensor with immobilized ADH. Transition metal oxides providing unique electrocatalytic properties and displaying strong interactions with noble metal nanoparticles were also employed for the construction of electrochemical platforms for ethanol biosensing. An illustrative example is the preparation of a mixed molybdenum and manganese oxide film electrode enriched with platinum nanoparticles for the preparation of a whole cell biosensor by immobilization of the intact *Gluconobacter oxydans* biofilm onto PtNPs/MnOx-MoOx/GCE and monitoring of oxygen consumption as a result of the bacterial metabolism in the presence of the substrate. The linear range found was 0.075–5.0 mM ethanol, with a response time of 63 s [33].

Most of the electrochemical biosensors designed for the determination of ethanol were applied to the analysis of alcoholic beverages (see Table 2). Applications to the analysis of biological samples are relatively scarce. An important reason for this is that ethanol itself is only measurable for a few hours after ethanol intake in biological matrices, including blood, urine, and sweat, these matrices being only useful to detect recent ethanol exposure. Because of this, since approximately early 2000, the non-oxidative ethanol metabolites have received increasing attention. Among these, ethyl *β*-d-6-glucuronide (EtG) stands out. This stable, nonvolatile, and minor direct-ethanol metabolite can be detected in urine from 6 h to 4 days after the last alcohol intake [34]. In an interesting article, Selvam et al. reported the detection and quantification of EtG in human sweat by using a label-free electrochemical chemi-impedance sensing method and designing a flexible and wearable sensor prototype. Gold or zinc planar electrodes were integrated on flexible polyimide, and monoclonal antibodies for the target compound were immobilized using thiol-based chemistry. Impedimetric measurements were made and calibrated for physiologically relevant doses of EtG over 1–10^4^ μg·L^−1^ (gold) or 0.001–100 μg·L^−1^ (ZnO) [35]. 

**Figure 2:**
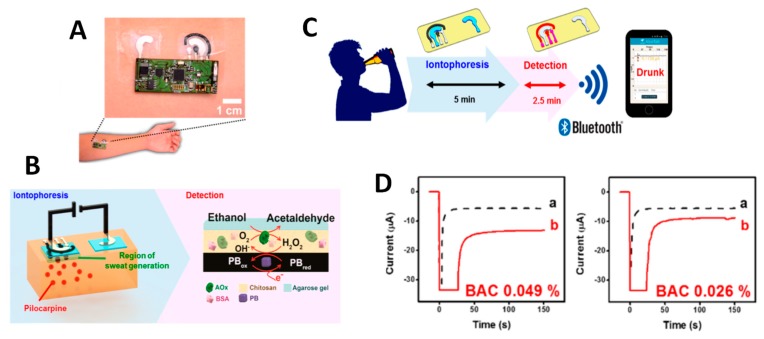
Alcohol iontophoretic-sensing tattoo device with integrated flexible electronics applied to a human patient (**A**); schematic diagram of constituents in the iontophoretic system (left) and processes involved in the amperometric sensing of ethanol (right) (**B**); scheme of the wireless operation for transdermal alcohol sensing (**C**); amperograms recorded before (a) and after (b) drinking alcohol beverage (**D**). BAC (blood alcohol concentration) recorded by a breath analyzer. Potential step to −0.2 V vs. Ag/AgCl. Reprinted from [38], with permission.

Other wearable biosensors for ethanol were described, enabling real-time, continuous, and fast detection, with a similar performance to that mentioned above [36,37]. For example, a wearable tattoo-based biosensing system was developed by Kim et al. [38] for noninvasive alcohol monitoring in induced sweat. The skin-monitoring platform (Figure 2) integrated a temporary tattoo system with an iontophoretic biosensor equipped with flexible wireless electronics. Moreover, transdermal delivery of pilocarpine drug induces sweat via iontophoresis, which is measured by amperometry, involving an AOx-coated screen-printed and Prussian blue (PB) electrode transducer. A lancet-free, label-free biosensor for the simultaneous determination of glucose and alcohol in sweat was prepared by using zinc oxide thin films integrated into a nanoporous flexible electrode system. Sensing was achieved from perspired human sweat at low volumes (1–3 μL), without external stimulation. Zinc oxide thin-film electrodes were surface functionalized with AOx, and alcohol monitoring was made by measuring impedance changes onto the sensor interface with a dynamic range between 0.01 and 200 mg·dL^−1^, with an LOD value of 0.01 mg·dL^−1^ ethanol [39]. 

Electrochemical instruments for health monitoring using smartphones or similar devices have arisen in recent years. Portability, real-time monitoring, and inexpensive measurements using techniques such as chronoamperometry, cyclic voltammetry, or EIS are the main features of these analytical tools. For instance, a smartphone-based μPotentiostat, combining sensor readout digitalization with a reusable lab-on-a-chip concept was developed for the determination of ethanol in whole blood [40]. According to the authors, direct blood measurements are advantageous compared to those involving breath sampling because of the greater immunity to errors, especially in the case of unconscious or noncollaborative patients. Biosensing was enabled by in situ electrodeposition of a calcium alginate hydrogel containing horseradish peroxidase (HRP) and AOx for selective ethanol detection. Then, 3,3′,5,5′-Tetramethylbenzidine (TMB) was used as the redox mediator, and amperometric measurements were performed at 0.0 V (vs. Pt pseudo-reference electrode). A calibration plot for ethanol, with a linear range up to 1.25 g·L^−1^ and a limit of quantification of 0.056 g·L^−1^ in blood, was obtained using only 40 μL of sample. 

### 2.3. Illicit Drugs

The abuse of medication or drugs is one of the most frequent causes for criminal and civil matters concerning addiction liability, personal injury, vehicle accidents, drug overdose, or murder [47]. Apart from alcohol and tobacco, other drugs of abuse are cannabinoids, cocaine, heroin, marijuana, amphetamine-related drugs, benzodiazepines, opioids, hallucinogens, such as lysergic acid diethylamide (LSD), and anesthetics, such as ketamine. These substances have different potential for abuse and, in some cases, legitimate medical uses. For example, heroin and LSD, as well as opioids, such as fentanyl, all have high potential for abuse, but opioids have medical but restricted use. Other drugs such as ketamine are currently accepted for medical use and have a moderate potential for abuse and low risk of dependence. As reviewed by Shaw and Dennany [48], electrochemical biosensors are a powerful tool in the forensic field for the analysis of these substances at low concentration in complex matrixes. Campuzano et al. also revised the use of electrochemical nucleic-acid-based biosensors for the determination of drugs of abuse and pharmaceuticals [49].

**Figure 3:**
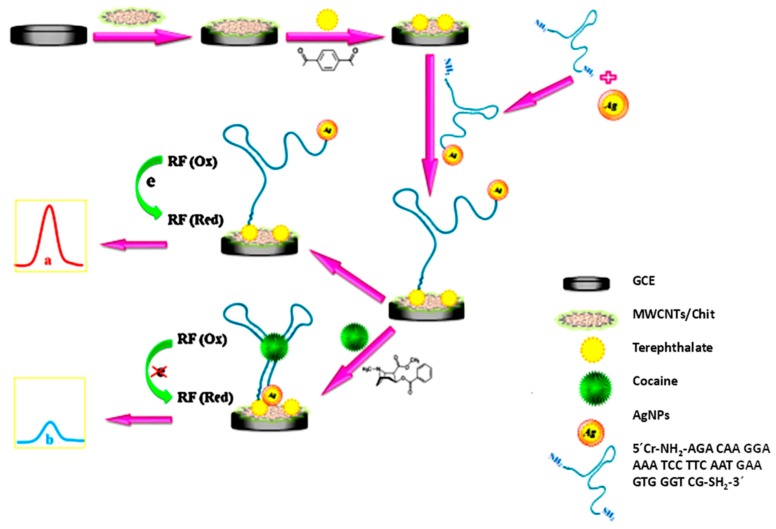
Schematic display of the preparation steps and functioning of the aptasensor constructed for cocaine detection. Reproduced from [52], with permission.

The challenging detection of trace concentrations of illicit drugs in forensic analysis was addressed in recent years by the use of affinity biosensors as an efficient alternative to more sophisticated and expensive techniques. Among the drugs of abuse, cocaine has received special attention, as it can be deduced from the high number of reported electrochemical biosensors, many of which involving aptamers [50]. A representative example is a label-free aptasensor using SPCEs modified with three-dimensional magnetic reduced graphene oxide (3D-MRGO)/polyaniline (PANI)/AuNPs composites for the impedimetric determination of cocaine. A specific thiolated cocaine aptamer was immobilized onto the modified electrode, and the analytical readout was obtained by measuring the increase in the R_CT_ in the presence of the target analyte [51]. In addition, immobilization of aptamer-functionalized AgNPs onto a nanocomposite prepared with MWCNTs, liquid ionic, and chitosan, and involving riboflavin as the redox probe, was employed for the construction of an electrochemical aptasensor for cocaine detection in human serum [52]. Figure 3 shows the steps involved in the preparation of the aptasensor. In the absence of cocaine (a) in the Figure 3, a well-defined DPV signal corresponding to the reduction of RF catalyzed by AgNPs was obtained. However, when introducing the target (b) in the Figure 3, it binds with aptamer in a three-way junction, giving rise to a steric restriction of the electrochemical reaction of RF, and the subsequent decrease in the peak current.

The main components of ecstasy tablets are 3,4-methylenedioxyamphetamine (MDA), 3,4-methylene-dioxymethamphetamine (MDMA), and 3,4-methylenedioxyethylamphetamine (MDEA). Monoclonal antibodies for amphetamine and methamphetamine (MA) were used to determine the respective antigens and methylenedioxy-analogues. Zhang and Qi [53] developed a label-free amperometric immunosensor using Prussian blue (PB) as an artificial peroxidase to detect MA. A hybrid of PtNPs and PB was co-deposited onto the electrode, which was further coated with a double-layer 2D-network of 3-mercaptopropyl-trimethoxy-silane (3-MPS) and AuNPs. Then, capture antibodies were immobilized, and the analytical signal related to the antigen concentration was monitored by the electrochemical H_2_O_2_ reaction catalyzed by PB. 

**Figure 4:**
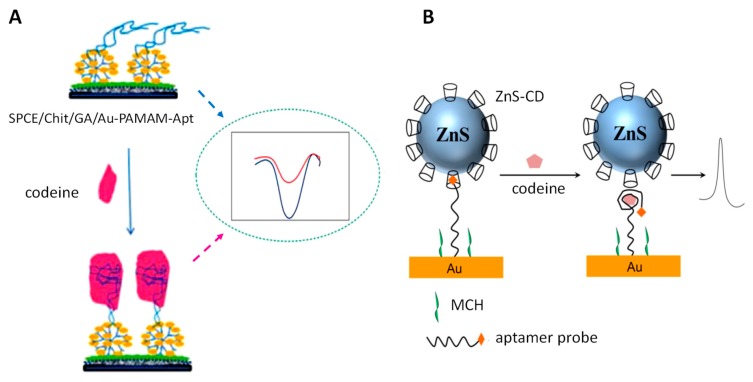
Schematic display of the fundamentals involved in the preparation of aptasensors for the determination of codeine. Reprinted from [55] (**A**) and [56] (**B**), with permission.

Electrochemical affinity biosensors were reported for the sensitive determination of morphine. An interesting example is the configuration prepared by Talemi and Mashhadizadeh [54], based on the intercalative and electrostatic interaction of morphine with ds-DNA immobilized onto mercapto-benzaldehyde-modified gold electrode. DPV was used as a transduction technique, and the determination of the alkaloid resulted feasible in a 0.05–500 μM range, as well as the application to urine and blood plasma. Another alkaloid separated from opium is codeine (3-methylmorphine), whose effects, although less strong than morphine, can also create a health risk. Among the methods described for codeine determination, those based on the interactions between codeine and binding aptamers can be highlighted. SPCEs modified with polyamidoamine dendrimers (PAMAM), glutaraldehyde, chitosan, and AuNPs (Figure 4A) were employed for the immobilization of the specific aptamer, and the analytical response was obtained by measuring the electron transfer decrease of [Fe(CN)_6_]^3−/4−^ probe by DPV [55]. Another interesting design involves a dually labelled DNA aptamer probe with dabcyl as an electrochemical tag and ZnS nanoparticles modified with cyclodextrins (ZnS-CDs), which interact with the codeine probe modified electrode through the host–guest recognition of CDs to dabcyl (Figure 4B). The addition of codeine provides aptamer folding, releasing ZnS-CDs into the solution and provoking an increase of the monitored voltammetric signal [56]. 

Tetrahydrocannabinol (THC) is the major component in marijuana affecting mental state and producing addiction, although, in addition, it has applications for the treatment of some diseases, such as multiple sclerosis or neurologic disorders [57]. THC can be determined using electrochemical biosensors, for instance, by preparing a double-layer AuNPs electrochemical immunosensor by immobilizing anti-THC antibody and using a chitosan/AuNPs/thionine/HRP amplification system. The amperometric response exhibited a linear correlation with the THC concentration from 0.01 to 10^3^ ng·mL^−1^, with an LOD of 3.3 pg·mL^−1^ [58]. Synthetic cannabinoids do not contain cannabis, but they are also included in the category of psychoactive substances because they produce similar effects when consumed and provoke several health events. The increase in the occurrence and the chemical diversity of these substances make it difficult to identify and monitor. Recently, an electrochemical biosensor for the determination of one of the synthetic cannabinoids, JWH-073 (also known as “Spice” or “K2”), was reported, using poly(methyl methacrylate) (PMMA) hyperbranched copolymer for the immobilization of the specific antibody. The calibration plot constructed by measuring the DPV peak current after addition of the target analyte showed a linear range between 25 and 500 ng·mL^−1^. The electrochemical biosensor was successfully applied for the target analyte in human urine [59].

### 2.4. Doping

The use of any illicit substance or method forbidden by the World Anti-Doping Agency (WADA) for enhancing athletic ability, training, and performance, is known as “doping” [60]. For decades, professional and elite athletes have widely used substances to improve their sport activities. The most common classes of doping drugs include not only illicit substances but also products sold as nutritional supplements. Anabolic steroids, peptide hormones, stimulants such as amphetamine, cocaine, caffeine, or ephedrine, narcotic analgesics and diuretics, among others [61]. Mass spectrometry (MS)–chromatographic techniques are currently mostly used for the determination of doping substances. However, beyond the high selectivity and sensitivity of these techniques, they are costly, time consuming, and require complex equipment, which is usually limited to laboratories. Therefore, due to the increasing need for faster, greener, and accessible point-of-care detection techniques, the use of biosensors is rising as a screening tool for the detection and quantification of doping substances in biological fluids. 

Hormones are the most potent and frequently used doping substances, and they responsible for approximately 2/3 of the detected violations of anti-doping rules. They occupy a prominent place in several categories of the WADA Prohibited List (https://www.wada-ama.org/sites/default/files/ wada_2019_english_ prohibited_list.pdf). Among them, the most representative are S1 (anabolic agents, mainly androgens), S2 (peptide hormones, growth factors, and related substances), S4 (hormone and metabolic modulators), and S9 (glucocorticoids). At present, the vast majority of positives are due to a wide variety of androgens, including commercialized and illicit (nutraceutical, designer) synthetic and exogenous natural androgens. Furthermore, peptide hormones, such as erythropoiesis stimulating agents, growth hormone, and its secretagogues, remain difficult to detect [62]. 

Dehydroepiandrosterone (DHEA) and dehydroepiandrosterone 3-sulfate (DHEA S) are androgen hormones used as doping substances because they can increase muscle mass and enhance strength. Recently, an electrochemical immunosensor involving gold surfaces modified with cysteamine where anti-DHEA S was immobilized via glutaraldehyde as a cross-linking agent was reported for DHEA analysis. Using DPV as the transduction technique, a linear range between 2.5 and 100 ng·mL^−1^ DHEA S was obtained. The immunosensor was applied to the analysis of synthetic serum and urine [63]. A sensitive immunosensor for testosterone was prepared using anti-testosterone nanobodies (Nbs) and electrochemical impedance spectroscopy (EIS). First, an immune Nbs library against testosterone was constructed, and, after biopanning, the Nb of the highest yield and stability was selected to couple with biotin in vivo. Then, a label-free immunosensor was implemented by immobilization of Biotin-Nbs onto a GCE modified with streptavidin. The determination was performed by measuring the charge transfer resistance variations against testosterone concentration, using [Fe(CN)_6_]^3−/4−^ as the redox probe. An LOD value of 45 pg·mL^−1^ was achieved [64]. 

**Figure 5:**
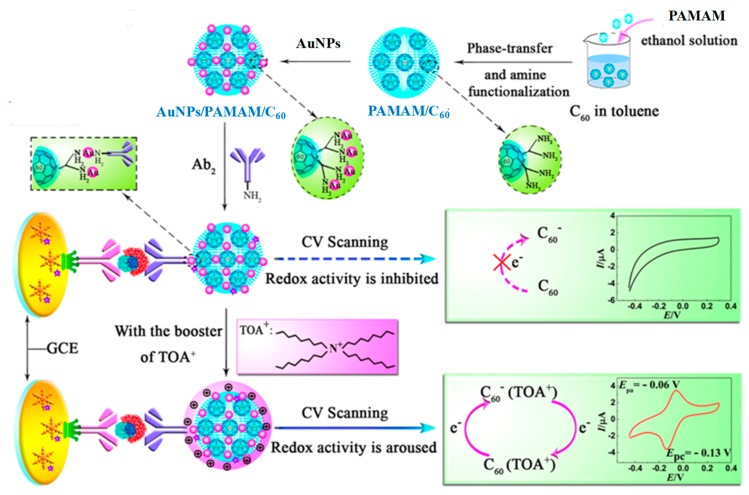
Scheme of the AuNPs/PAMAM/C_60_ synthesis used as nanocarrier in the preparation of an immunosensor for the determination of EPO, and possible mechanism of the electrochemical reaction used to monitor the affinity reaction. Reprinted from [66], with permission.

Erythropoietin (EPO) is the most important peptide hormone used as a blood-doping agent. It stimulates the production of new red blood cells, and, therefore, athletes use EPO illicitly to enhance their performance by boosting the delivery of oxygen to the tissues [65]. Regarding recent electrochemical biosensors, only one paper was found that deals with EPO monitoring in human serum. The biosensor is a sandwich-type immunosensor involving the use of fullerene (C_60_) was functionalized with PAMAM and gold nanoparticles, AuNPs/PAMAM/C_60_, as nanocarrier to label detection antibodies for EPO. Figure 5 shows that the capture antibody was immobilized onto a GCE modified with a thin layer of nanodendrites and protein A. After the addition of tetraoctyl bromide (TOAB), which acts as a booster to arouse the inner redox activity of C_60_ conjugates, the electrochemical response was obtained by cyclic voltammetry. The resulting immunosensor provided a linear calibration plot over the 0.01 to 80 mIU mL^−1^ EPO range [66].

Over the years, the abuse of human growth hormone (hGH) by athletes, leading to performance-boosting effects, has been reported. An electrochemical immunosensor involving a GCE modified with flowerlike diphenylalanine peptide nanostructures (FPNSs) was developed for the determination of this hormone in human serum. Antibodies were covalently immobilized onto the surface of FPNSs, and the measurement of R_CT_ provided a linear detection range of 1–100 pg·mL^−1^ hGH [67]. Another approach used for the detection of hGH abuse is the monitoring of hGH biomarkers. For instance, insulin-like growth factor-I (IGF-I) was reported to be a prominent biomarker of hGH administration [68], and various electrochemical immunosensors were reported for its determination. In a simple strategy, a label-free configuration was prepared by immobilizing anti-IGF-1, using MWCNTs and an ionic liquid. The variation of the R_CT_ was linear with the logarithm of the IGF-1 concentrations between 0.4 and 15 ng·mL^−1^, with an LOD of 22 pg·mL^−1^ [69]. 

The administration of glucocorticoids by oral, intravenous, intramuscular, or rectal routes is prohibited in sports competitions. Synthetic glucocorticoids are derived from cortisol, the endogenous glucocorticoid produced in the adrenal glands. A fast and reliable monitoring of cortisol in saliva was achieved with a high-performance field-effect transistor (FET)-based biosensor constructed from N-doped multidimensional carbon nanofibers. Anti-cortisol antibodies were immobilized onto the conductive channels of FET, and changes associated to cortisol concentrations were measured in a wide range, from 100 aM to 10 nM [70]. A paper-based biosensor chip was also fabricated for the detection of salivary cortisol. First, spin-coating of a graphene nanoplatelets and amphiphilic polymer composite was made onto Whatman filter paper, and then micro gold electrodes were deposited. The resulting platform was incubated with a mixture of dithiobis(succinimidyl propionate) (DTSP) and NaBH_4_, to form a self-assembled monolayer (SAM), where the capture antibodies were covalently attached, followed by blocking of the remaining activated groups by BSA. Using EIS as the electroanalytical technique, a low LOD value of 0.87 pg·mL^−1^ was reported [71]. Dexamethasone (DXN) is a synthetic hormone that belongs to the group of corticosteroids, which is often used as a growth-promoting agent to increase the body mass. Recently, a high-specificity aptamer-ligand biorecognition and binding system was reported to monitor DXN. The detection principle was based on a label-free electrochemical aptasensor, involving immobilization of an aptamer designated as DEX04 onto a gold electrode, making possible the development of an impedimetric aptasensor based on the measurement of the R_CT_ of the [Fe(CN)_6_]^4−/3−^ redox couple. The aptasensor exhibited a linear range from 2.5 to 100 nM, with an LOD of 2.12 nM [72].

Diuretics were first banned in sport in 1988 due to their use by athletes to eliminate water from the body, causing a rapid weight loss in order to comply with weight limits in sports, such as boxing, judo, and weight lifting, as well as to mask the administration of other doping agents by reducing their concentrations in urine. Acetazolamide (ACTZ), an inhibitor of the carbonic anhydrase enzyme, is one of the diuretics used by athletes, although it can cause arrhythmia or dehydration, among other health disorders. A bio-inspired electrochemical sensor using a binuclear oxo manganese complex exhibiting biomimetic activity, according to the Michaelis–Menten model, and good catalytic properties in the oxidation of ACTZ was applied to the detection of the diuretic in real urine samples for doping control analysis. The characteristics of the complex are similar to the active sites of enzymes involving manganese cofactor, as these provide the high selectivity and sensitivity. Furthermore, the high stability was the most important property of this configuration. Indeed, the same electrode surface could be used for more than 500 separate determinations [73].

### 2.5. Toxins

Toxin pollution is one of the most important issues for food safety guarantee. It has been stated that, to some degree, up to 25% of the world’s agricultural commodities become contaminated by mycotoxins produced by filamentous fungi during crop growth, harvest, storage, or processing [74]. Deoxynivalenol, zearalenone, nivalenol, ochratoxin A (OTA), aflatoxin B1 (AFB1), and fumonisin B1 (FB1) are some of the most predominant mycotoxins [75]. Among them, AFB1 is listed as group I carcinogens by the International Agency for Research on Cancer [76] and is claimed as the most toxic mycotoxin due to its capacity to bind with the DNA of cells increasing the risk of liver cancer in human beings [77]. The US Food and Drug Administration (FDA) has set the limited level of AFB1 in corn and peanut feeds for finishing beef cattle at 300 ng·mL^−1^ [78]. In recent years, because of the need to detect this and other mycotoxins at very low concentration levels, many electrochemical biosensors involving different configurations and materials were reported. Table 3 summarizes the main characteristics of some recent methodologies, and, hereinafter, a few relevant examples are used to illustrate their usefulness.

**Figure 6:**
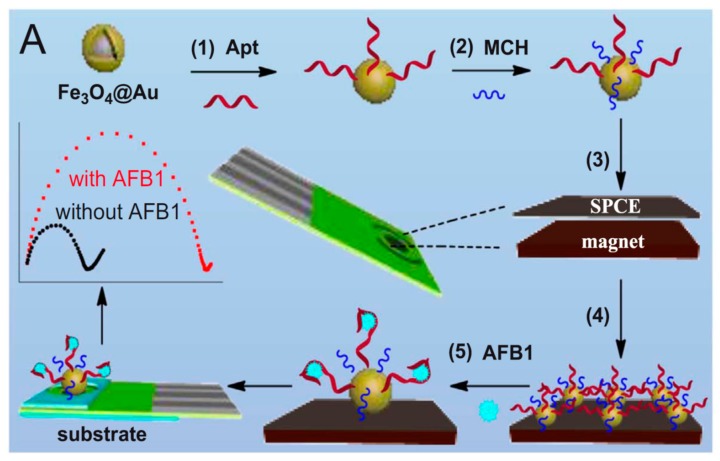
Schematic illustration of the fabrication of a magnetically assembled aptasensor for AFB1. Reprinted from [79], with permission.

Wang et al. reported a magnetically assembled aptasensing device for label-free determination of AFB1 by employing a disposable SPCE coated with a designed polydimethylsiloxane (PDMS) film as a microelectrolytic cell (Figure 6). The determination of AFB1 was performed by EIS upon aptamer-target biorecognition. The developed method provided a linear calibration extending over the 20 to 50 ng·mL^−1^ range, with an LOD of 15 pg·mL^−1^ (S/N = 3), and was applied to the analysis of spiked peanuts [79]. Another electrochemical aptasensor was developed for the detection of aflatoxin M1 (AFM1), using an AFM1 aptamer and AuNPs. The fundamentals of the detection rely on conformational changes of hairpin structure of the aptamer (Apt), in the presence and absence of AFM1. Once the Apt is immobilized onto SPAuE, a complementary strand (CS), conjugated with AuNPs, comes to close proximity of Apt-SPAuE. In the presence of AFM1, the hairpin structure of Apt is lost to form the Apt-AFM1 complex, and the 5′ end of Apt hybridizes with CS. The addition of methylene blue (MB) as redox agent provoked its electrostatic accumulation on the electrode surface, with AuNPs giving rise to a strong current signal. The aptasensor allowed determination of AFM1 with an LOD of 0.9 ng·L^−1^ and was successfully applied in real samples, including milk and serum [80].

**Figure 7:**
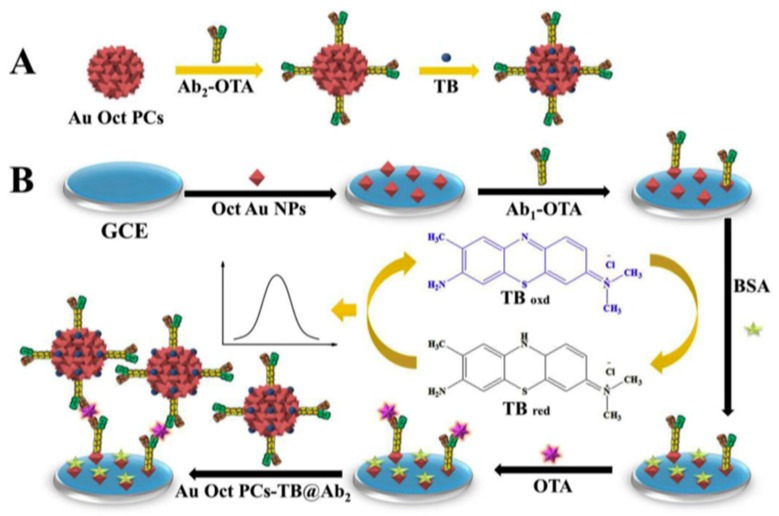
(**A**) Preparation process of AuOCtPCs and (**B**) fabrication steps of the immunosensor for OTA detection. Ab_1_ and Ab_2_ capture and detector OTA antibodies, respectively. Reproduced from [84], with permission.

Ochratoxins are dangerous by-products mainly produced by several species of storage fungi, including the penicillium and aspergillus [81]. OTA was identified as one of the most toxic and carcinogenic substances for a wide variety of mammalian species [82]. A variety of agricultural products, including wheat, corn, barley, coffee, fruit, and rice, can be easily contaminated by OTA. The European Commission established some regulatory limits to control OTA levels and, for example, the maximum tolerated level for raw cereal grains is 5 ppb [83]. Among the numerous methods for OTA biosensing, it is worth mentioning the preparation of an electrochemical immunosensor involving octahedral plasmonic colloidosomes (AuOctPCs) as substrates for the immobilization of specific OTA antibodies and as labels for signal amplification [84]. Octahedral gold nanoparticles (OctAuNPs), obtained by the reduction of HAuCl_4_ in the presence of PDDA (poly(diallyldimethylammonium) and ethylene glycol, were used to immobilize the capture antibody onto the electrode surface. Furthermore, AuOctPCs were prepared by natural settlement of OctAuNPs in 1-butanol/water reversed-phase emulsion (Figure 7). The resulting nanomaterial, with edges and sharp corners, exhibited high specific surface area and good electron transfer ability, allowing the immobilization of a great amount of antibodies; it was also able to interact with the redox mediator toluidine blue (TB), acting as a carrier tag for current enhancement. Using square wave voltammetry (SWV), the proposed immunosensor provided a linear calibration range from 0.1 to 10^4^ pg·mL^−1^ and an LOD value of 39 fg·mL^−1^. 

In addition to mycotoxins, other products derived from some foods are of interest due to their possible adverse effects on health at very low concentration levels. An example are toxic microalgae species, which contaminate shellfish, producing various forms of human poisoning that is considered a relevant global problem due to the worldwide distribution of these toxins. Saxitoxin (STX) and its analogous cause paralytic shellfish poisoning (PSP), blocking sodium transport through sodium-channel receptors. Therefore, sensitive and robust methods for their detection in complex samples must be developed. The use of electrochemical biosensors for the analysis of marine toxins was reviewed by Liang et al. [85]. In a relevant paper, a dithiol SAM-based immunoassay was reported, using gold electrode arrays for the construction of an electrochemical immunosensor for tetrodotoxin (TTX), yielding an LOD of 2.6 ng·mL^−1^. The applicability of the method was demonstrated by TTXs quantification in different tissues of several puffer fish species, at levels as low as 0.07 mg TTX equiv. kg^−1^ tissue, well below the Japanese limit value of 2 mg TTX equiv. kg^−1^ tissue used as a criterion to consider puffer fish safe for consumption [86]. A miniaturized potentiometric saxitoxin immunosensor was reported, involving graphene nanosheets with incorporated lipid films and immobilized anti-STX. The achieved LOD was 1 nM, and the method was tested in lake water and shellfish samples [87].

Okadaic acid (OA) is a marine lipophilic toxin produced by toxicogenic dinoflagellates. OA may accumulate in the digestive glands of shellfish when they feed on these kinds of microalgae. It is the major diarrheic shellfish poisoning (DSP) toxin in humans, since OA causes blocking of the active sites of enzymes and consequently inhibits serine/threonine protein phosphatases type 1 (PP1) and type 2A (PP2A), resulting in an over-phosphorylation of proteins in cells and gastrointestinal troubles. The European Commission Regulation EU No 786/2013 establishes a maximum permitted concentration of 160 μg OA per kg of live bivalve mollusks for human consumption [88]. Based on the inhibition of protein phosphatase 2A (PP2A) by OA, an electrochemical enzyme biosensor which involved SPCEs modified with an electropolymerized poly-o-aminophenol (PoAP)/CNTs composite film for the enzyme immobilization was reported. After incubation of OA standard solutions or the samples onto PoAP/PP2A/CNTs/SPCE, the addition of *p*-nitrophenol phosphate (*p-*NPP) allowed the DPV quantification of OA within a 1–300 μg·L^−1^ linear range, with an LOD value of 0.55 μg·L^−1^ [89]. 

**Figure 8:**
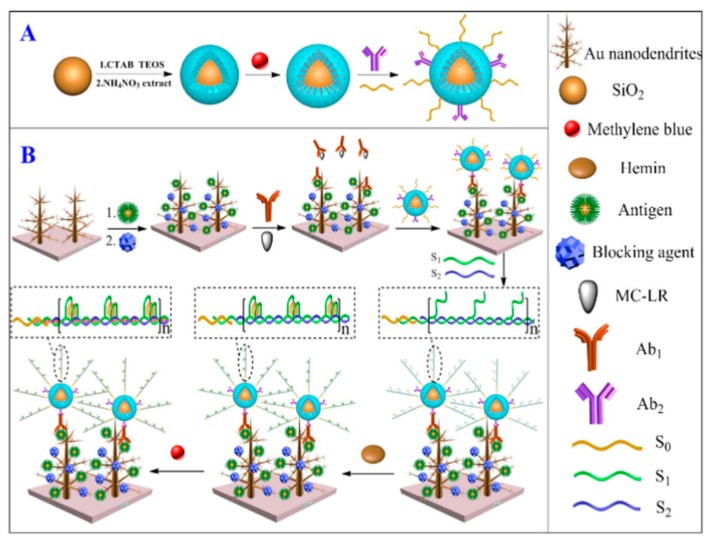
Schematic illustration of the preparation of SiO_2_@MSN-NH_2_-MB-Ab_2_-S_0_ nano-biomaterial conjugation (**A**) and the construction of the electrochemical immunosensor for MC-LR (**B**). Reproduced from [92], with permission.

Another family of toxins causing severe human health problems due to their hepatotoxicity and tumor-promoting activity are microcystins (MCs) [90]. Among these, microcystin-LR (MC-LR), produced by cyanobacteria, is one of the most toxic. Because of their sensitivity and relative simplicity, aptasensing strategies have received great attention for the detection of MC-LR [91]. Gan et al. reported a multiple amplified enzyme-free biosensor for MC-LR detection using G-quadruplex/hemin functionalized mesoporous silica with redox-active intercalators [92]. Figure 8 shows the synthesis of monodisperse core-shell mesoporous silica (SiO_2_@MSN)-functionalized DNAzyme concatemers to load hemin and MB as the mimic enzyme. A secondary antibody (Ab_2_) able to recognize the MC-LR antibody (Ab_1_) and a DNA strand as the initiator were immobilized, whereas two auxiliary DNA strands were used for the in situ propagation to form a double-helix DNA through hybridization chain reaction (HCR), forming numerous DNAzymes (G-quadruplex/hemin) after the addition of hemin. The intercalation of MB/DNA improved the catalytic ability of DNAzymes toward the reduction of H_2_O_2_ as electrochemical readout. This configuration could detect MC-LR in a 0.5 to 25 μg·L^−1^ range, with an LOD value of 0.3 ng·L^−1^. 

### 2.6. Microorganisms

Infectious agents are responsible for diseases throughout the world caused by contaminated water, food intoxication, hospital-acquired, and pandemics. Pathogenic bacteria pose serious problems for public health and provoke significant economic losses. Early detection is difficult because the standard analytical methods involve complex handling processes, expensive instruments, and qualified experts. These drawbacks have led to efforts for the development of sensitive, specific, robust, and fast methods to facilitate reliable results, and, in this context, electrochemical biosensors can be considered relevant tools. 

Among foodborne bacterial pathogens, *Escherichia coli* (*E. coli*) serotype O157:H7, causes severe diseases in humans. An electrochemical biosensor for its detection was constructed by interfacing graphene nanostructures functionalized with specific antibodies able to immobilize bacteria on the sensor surface. The developed device provided non-faradaic electrochemical responses related to the number of cells per mL, with no need for redox probe, and allowed the detection of bacteria to as low as 10–100 cells mL^−1^ [114].

**Figure 9:**
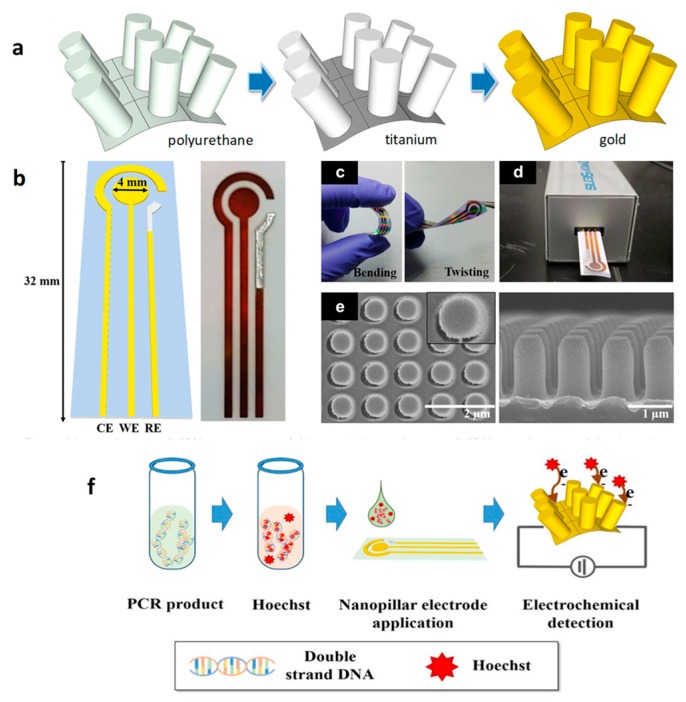
Illustration of the steps involved in the preparation of flexible nanopillar electrodes (NPE) steps (**a**); scheme (**b**) and photographic images of bending and twisting (**c**); USB connection (**d**); and SEM of top and side view of NPE (**e**). NPE-based electrochemical evaluation of *E. coli* O157:H7 (**f**). Reprinted from [115], with permission.

Fabrication of flexible electrochemical platforms constitutes, nowadays, a research line of growing interest for the easy implementation and the variety of applications. Among them, it is worth mentioning the importance of these systems as point-of-care testing (POCT) devices for the continuous monitoring of foodborne diseases [115]. An illustrative example is a flexible and highly ordered nanopillar array prepared with gold and silver electrodes which exhibits an excellent electrochemical performance to detect the PCR amplified gene from *E. coli* O157:H7. As Figure 9 shows, thin titanium and gold layers were prepared by vacuum sputtering on the surface of nanopillar arrays. The as-prepared gold electrodes were used as working and counter electrodes, and silver was further printed to be used as the reference electrode. For the electrochemical detection, the amplified gene of *E. coli* O157:H7 was mixed with the Hoechst electrolyte, which specifically intercalates with dsDNA, and SVW was employed as the transduction technique. A linear detection range from 10 to 10^5^ colony-forming units (CFUs) was achieved, and the biosensor was applied to the analysis of milk samples.

*Listeria monocytogenes* is transmitted to humans when food comes into contact with contaminated water or soil. The presence of this microorganism is a persistent problem since it can proliferate under conditions of low moisture, high salinity, or common freezer temperatures. Hill et al. [116] developed a sensing strategy for the rapid detection of *L. monocytogenes* in food samples which involved the preparation of chitosan-aptamer (or antibody) nanobrush borders immobilized onto Pt/Ir electrodes modified with reduced graphene oxide and platinum nanoparticles. The selective capture of bacteria and the detection steps were implemented on the basis of the pH-dependent stimulus-responsive chitosan nanobrushes decorated with receptors that bind a cell surface target. Cells were captured onto extended nanobrushes at pH < 6, while impedimetric measurements were made at pH > 6, where nanobrushes collapsed. The biomimetic material was used to develop a rapid test (17 min) for selectively detecting *L. monocytogenes* from 9 to 10^7^ cfu·mL^−1^, with no preconcentration, and in the presence of other gram-positive cells. 

Integration of sensors and microfluidics constitutes the fundamentals of lab on a chip technology, which has demonstrated its usefulness in diverse fields, such as food safety monitoring. In this regard, a miniaturized portable EIS platform was prepared for the detection of *L. monocytogenes* in milk. It consisted of a microfluidic device with EIS sensors connected to a portable impedance analyzer for on-field application. An array of interdigitated microelectrodes functionalized with specific antibodies was used, providing a linear dependence between the charge transfer resistance and the bacteria population over the 100 to 2200 CFUs·mL^−1^ range [117].

Another widespread foodborne pathogen is *Salmonella*, a gram-negative rods genus belonging to the *Enterobacteriaceae* family, for which over 2500 different serotypes or serovars were identified to date. *Salmonella* is a ubiquitous and hardy bacterium that can survive several weeks in a dry environment and several months in water (see https://www.who.int/news-room/fact-sheets/detail/salmonella-(non-typhoidal)). Furthermore, *Salmonella* is a ubiquitous and hardy bacterium that survives several weeks in a dry environment and several months in water. Lu et al. developed an electrochemical immunosensor for the determination of *Salmonella* in milk, using a double-layer AuNPs electrode for immobilization of the plasmid virulence C (*S*pvC) antibody. The electrochemical response was amplified by enhancing the number of immobilized antibodies and implementing a system for signal amplification, involving AuNPs-thionine-chitosan adsorbing HRP. The detection strategy implied that the amperometric measurement derived from the electrochemical reaction of H_2_O_2_ catalyzed by HRP in the presence of TH as the redox mediator. This scheme allowed determining *Salmonella* within a range of 10 to 5 × 10^4^ CFUs·mL^−1^, with an LOD value of 5 CFUs·mL^−1^ [118]. 

*Clostridium perfringens* must also be cited as one of the most common foodborne pathogens. The predominant pathogen is a spore-forming, rod-shaped, gram-positive bacterium widely found in different environments and foods types and associated with two kinds of foodborne diseases: diarrhea and enteritis necroticans [119]. *C. perfringens* could be detected with a label-free electrochemical DNA biosensor constructed by immobilization of the DNA probe onto a CeO_2_/chitosan-modified GCE. Under optimal experimental conditions, electrochemical impedance measurements were selective toward target DNA in comparison with base-mismatched and noncomplementary DNA. The dynamic range for detecting the target oligonucleotide sequence of *C. perfringens* was 10^−14^–10^−7^ mol·L^−1^ [120]. 

## 3. Electrochemical Biosensors for Chemical and Biological Weapons

The detection of explosives, especially 2,4,6-trinitrotoluene (TNT), attracts worldwide interests because of the threats for public security, as well as for human health, since they have become pollutants in natural water and other environmental samples. The development of rapid, cost-effective, and reliable assays for the detection of these molecules in both aqueous and gaseous samples is a high priority for forensic investigators, counterterrorism agencies, and global de-mining projects. Some biosensors were described for determining explosives, including electrochemical biosensors because of their outstanding sensitivity and selectivity using aptamers, peptides, or antibodies. Moreover, bio-inspired sensors involving the design of bio-mimic-recognized components, such as molecularly imprinted polymers, are also used for this purpose [121]. An interesting example is an electrochemical aptasensor for the determination of TNT involving the use of AgNPs and thiol-functionalized graphene quantum dots nanocomposite onto GCE and rutin as redox probe [122]. The same group prepared other biosensing surfaces for this nitroaromatic explosive [123]. Aptasensing and molecular imprinting was combined in an original configuration to prepare a nanohybrid receptor. An amino-aptamer and TNT were mixed and covalently bound onto an AuNPs@fullerene C_60_-modified GCE, followed by dopamine electropolymerization. After TNT removal, the cavity and the aptamer acted synergistically to recognize the target explosive. This strategy provided a wide linear concentration range by impedimetric measurements (0.01 fmol·L^−1^–1.5 μmol·L^−1^) and a very low LOD value of 3.5 amol·L^−1^ [124].

Peptide biosensors involve the use of short amino acid chains designed according to the binding sites of antibodies and are chemically synthesized to mimic specific molecular recognition. Their main advantage is the stability and possibility of long-term storage. Making use of this strategy, Zhang et al. [125] developed an impedimetric biosensor for TNT monitoring, using a specific peptide and combining the resulting platform with a smartphone. Electrodes were functionalized with the peptide, and the response was collected by a hand-held device and transferred wirelessly to a smartphone via Bluetooth connection. The method allowed for the displaying of TNT concentrations as low as 10^−6^ M in real time. Another configuration for the detection of TNT is a label-free peptide aptamer (peptamer) in which a ternary assembly layer consisting of anti-TNT peptamer, dithiothreitol (DTT), and 6-mercaptohexanol (MCH), forming Au/peptamer–DTT/MCH, was used. A linear relationship between the change in R_CT_ and the logarithm of TNT concentration was achieved from 0.44 to 18.92 pmol·L^−1^, with an LOD of 0.15 pmol·L^−1^. The ternary assembly layer provided an OH-rich hydrophilic environment and a highly compact surface layer which reduced the non-covalent binding (physisorption) of the peptamer and the nonspecific adsorption of TNT onto the electrode surface, thus leading to a high sensitivity [126]. 

Ion-selective field effect transistors (ISFET) conjugated to biorecognition molecules were also proposed for the detection of explosives. An example is a fully depleted silicon-on-insulator-based ISFET highly sensitive to changes in the gate solution, where *E. coli* nitroreductase was covalently attached, as the recognizing element, used for the detection of nitroaromatic explosives. The enzyme-catalyzed reduction of the analytes was conjugated to the oxidation of NADPH to NADP^+^ and the drain current induced by the reaction, which increased in the presence of the explosive, was measured. In the case of TNT, the dynamic range of the analytical response ranged between 10^−7^ and 10^−5^ mol·L^−1^. In addition, this biosensor was combined with a microfluidic system for analyte delivery and applied to the determination of explosives in water samples [127]. 

### 3.1. Chemical Warfare Agents (CWAs)

**Figure 10:**
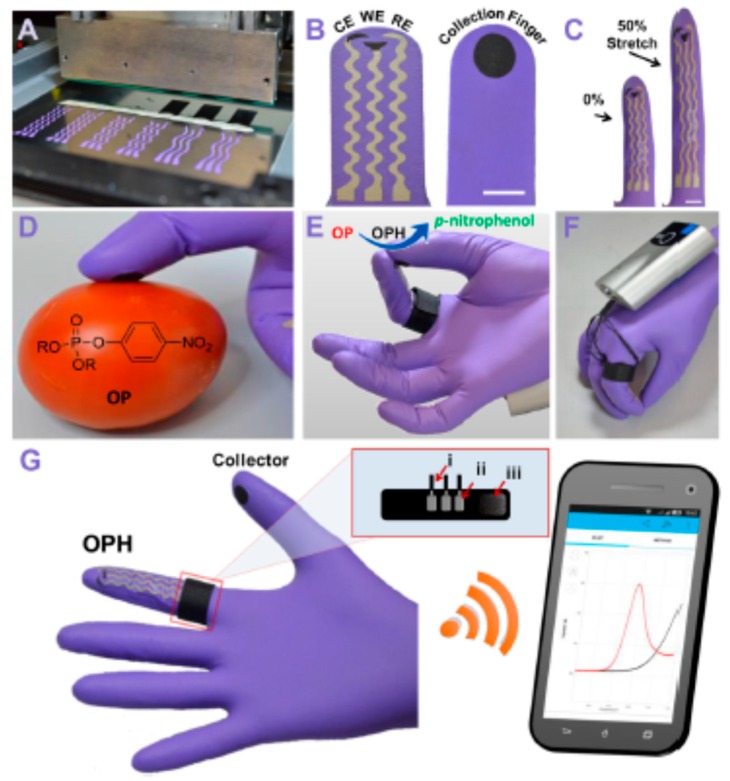
Scheme of the fabrication and functioning of a flexible glove biosensor to detect nerve agents OPs. (**A**) serpentine stencil design printing; (**B**) biosensing scan index finger (left) with CE, WE, and Ag/AgCl RE electrodes, and collecting thumb finger with printed carbon pad; (**C**) biosensing index finger under 0% and 50% linear stretch; (**D**) sampling chemical threat residues; (**E**) on-glove sensing procedure by joining the index and thumb fingers to complete the electrochemical cell; (**F,G**) photographs of the wearable glove biosensor connected to the portable potentiostat with wireless communication to a smartphone. Reproduced from [132], with permission.

CWAs are low-molecular-weight synthetic compounds characterized by being fast-acting, and sometimes lethal, even at low levels [128]. A variety of species belong to this group, including gaseous blood or chocking agents, volatile nerve and blister agents, nonvolatile vomiting agents, and nonvolatile lachrymators (tear gases). Recently, a wearable origami-like paper-based electrochemical biosensor for the determination of sulfur mustard (commonly known as mustard gas) directly in the aerosol and liquid phase was reported [129]. The electrodes were prepared by screen-printing onto a filter-paper support, and the detection was based on the inhibitory effects toward the enzymatic oxidation of choline catalyzed by choline oxidase, followed by detection of the reaction product, H_2_O_2_. Apart from the enzyme, the conductive graphite ink of the working electrode was also modified with carbon black/PB nanocomposite to electrocatalyze the H_2_O_2_ reduction. The resulting biosensor allowed the fast detection of real sulfur mustard with an LOD value of 0.019 g.min m^−3^ for aerosol phase. Inhibition of enzymatic reaction was also employed for the detection of other CWAs, such as organophosphate derivatives (OP). An interesting example is an electrochemical biosensor involving MWCNTs and acetylcholinesterase for the determination of paraoxon with an LOD value of 0.1 nM [130]. A sensitive amperometric acetylcholinesterase biosensor for OPs, using a 4,7-di (furan-2-yl) benzo [1,2,5] thiadiazole polymer, AgNPs and amine-functionalized rGO, was recently reported, as well. A linear range between 0.0206 and 2.06 μg·L^−1^ trichlorfon and an LOD value of 1 ng·L^−1^ were reported [131]. Mishra et al. [132] proposed a disposable glove-based sensing platform to detect toxic chemicals, including nerve agents OP compounds. Figure 10 shows as the flexible and wearable “lab-on-a-glove” integrated the enzyme immobilized on the index finger (detection finger), as well as the thumb (sampling finger) used to collect OP residues. The electrochemical reaction was completed when the thumb was joined with the sensing (index) finger, and a conductive semisolid gel matrix containing organophosphate hydrolase (OPH) was added. Then, the electrochemical response was wirelessly transmitted to a smartphone. Various target surfaces contaminated with OP compounds (methyl paraoxon and methyl parathion) were successfully assayed. 

### 3.2. Biological Weapons

Biological toxins are molecules produced by living organisms that induce harmful effects in other organisms by inhalation, ingestion, injection, or absorption. These toxins play a relevant role in the health and the security sectors. Some of these substances can be involved in natural intoxications, as they are the case of botulinum neurotoxin (BoNT), but the high toxicity and practical absence of antidotes has led to their classification as potential bioterrorism agents [133]. Worryingly, typical biological warfare agents (BWAs), such as *Bacillus antracis*, *Brucella* sp., *Yersinia pestis*, and Staphylococcal enterotoxin B, or the already cited BoNT, can be produced and spread not only by military but also by terrorist groups [134]. Therefore, the development of analytical tools that provide a means for the rapid and in situ detection of these toxins or bacteria, is widely claimed and electrochemical biosensors can be a valuable aid in this field. As a relevant example, a nanocomposite film consisting of AuNPs/graphene-chitosan was used to construct an impedimetric immunosensor for detection of botulinum neurotoxin A (BoNT/A). The specific BoNT/A antibody was immobilized onto the modified GCE, and the impedance changes due to the restricted electron transfer of redox probe in the presence of the antigen were employed as analytical readout. The toxin was determined in milk and human serum across a 0.27–268 pg·mL^−1^ range, with an LOD of 0.11 pg·mL^−1^ [135]. Mazzaracchio et al. [136] also prepared an impedimetric biosensor for *Bacillus anthracis* spore simulant (*B. cereus* spore) determination by immobilizing onto gold screen-printed electrodes a specific ssDNA aptamer as the recognition element and measuring the increase in R_CT_ after binding *B. cereus* spores with the aptamer. The linear range for the determination extended from 10^4^ to 10^6^ cfu·mL^−1^, and the LOD value was 3 × 10^3^ cfu·mL^−1^. A DNA electrochemical biosensor for the detection of *Bacillus anthracis*, based on a stem-loop probe, was also described, in which repetitive, fast, and versatile “on-off” signaling was performed. The DNA molecular beacon probe was immobilized onto a gold electrode, in its folded state, through an alkanethiol linker at the 5′ end, while methylene blue, as the redox label, was in the 3′ end. In this case, a 22.9–86.0 nM linear detection range was found [137].

Cholera toxin (CT) is a protein enterotoxin that is biologically active and interacts with specific gangliosides in natural and artificial membranes. Secreted by the bacterium *Vibrio cholerae*, it causes epidemic diseases that lead to rapid dehydration and death. Since CT fits the bioterrorism profile, there is increasing interest in the development of rapid and sensitive methods for its detection. Karapetis et al. developed a miniaturized potentiometric sensor with immobilized Ganglioside GM1, the natural cholera toxin receptor, onto stabilized lipid films on graphene nanosheets. The as-prepared biosensor allowed the detection to be performed over a wide range of toxin concentrations, providing a fast response time of ca. 5 min, and an LOD of 1 nM [138]. Finally, as an example of a toxin from vegetal origins, it is worth mentioning ricin, from the plant *Ricinus communis*, which is a cytotoxic protein that is considered to be a potential threat agent for terrorist use because of its high toxicity, absence of curative treatments, and ease of manufacture. Various immunosensors for the determination of this toxin are reported in the literature. An interesting example implies the use of single-domain antibodies (nanobodies) as recognition elements. Gold interdigitated electrodes (IDEs) modified with DTSP SAM were used for antibody immobilization. The immunosensor provided a linear CV current vs. log ricin concentration over the 1 fg·mL^−1^ to 1 μg·mL^−1^ range [139].

## 4. Conclusions

The unique opportunities provided by electrochemical biosensors to perform reliable determinations at the point of attention of analytes at different molecular levels and in samples of a very different nature justify their great potential and usefulness in many relevant fields. Although forensic analysis is not one of the fields where electrochemical biosensors were widely exploited, the applications highlighted and discussed in this review article confirm their great versatility and utility for the determination of a wide variety of toxic substances, including inorganic (arsenic and cyanide), organic (ethanol), illicit drugs and doping agents, toxins and microorganisms, chemical (explosives and CWAs), and biological weapons. When applied to the resolution of forensic problems, electrochemical biosensors reveal important advantages, such as low detection limits, wide linear response range, and good reproducibility. In addition, the proper modification of the electrode surfaces can improve these advantages, making it possible to design biosensors with the desired features. For instance, chemically modified electrodes have gained attention in the development of biosensors for drug analysis, owing to the simple surface renewal and the wide window of accessible potential.

Enzyme and whole-cell biosensors, as well as affinity sensors, mainly aptasensors, but also immunosensors and, more scarcely, nucleic acids or peptides biosensors, were used to solve a variety of analytical problems related to forensic practice. The enzymatic activity of enzymes or whole cells, and selective enzymatic inhibition phenomena, was exploited for the sensitive and almost specific determination of inorganic toxics, ethanol, toxins, and CWAs. Enzymatic biosensors involve mostly amperometric but also potentiometric transduction. It is worth remarking that aptasensors are nowadays efficient alternatives for determining toxics, drugs, and doping agents, as well as toxins and microorganisms. They are usually employed in connection with displacement assays in label-free approaches, using EIS or DPV transduction in the presence of selected redox probes. So far, immunosensing strategies in the forensic field were mostly employed for the determination of doping agents and toxins involving direct or sandwich formats coupled with label-free or label-based detection approaches, respectively. It is also important to mention that, in order to achieve the required sensitivity, many of the reported strategies make use of a wide variety of nanomaterials (CNTs, metallic NPs, and transition metal oxides) as electrode modifiers, both individually and in combination, to improve the electrocatalytic properties toward the analytes involved in the enzymatic/electrochemical reactions, as well as advanced labels or carrier tags. Other amplification strategies, such as hybridization chain reaction (HCR) and exonuclease catalyzed reaction can be profited. 

However, despite the demonstrated versatility of design and usefulness, it is necessary to be aware that the applications of electrochemical biosensors in forensic analysis are less advanced than in other fields, and proper attention should be paid to show their potential in the analysis of biological fluids and to perform multiplexed determinations. Indeed, an aspect to reinforce is the usefulness of electrochemical biosensors for the analysis of the real complex samples that constitutes the final objective of forensic analysis, with full guarantees of accuracy and precision. Despite the long way ahead to exploit the full potential of electrochemical biosensors in this amazing field, recent developments of biodevices involving the use of artificial (molecular imprinted polymers) or new bioreceptors (nanobodies and DNAzymes), implemented in wearable tattoo-based and flexible substrates or integrated on smartphone-based μ-potentiostat or microfluidic systems, show fairly well the progresses of electrochemical biosensors in forensic science. Bearing in mind that the advance of electrochemical biosensors in forensic science seem to follow a trend similar to that already experienced in other areas, such as clinical diagnosis, it makes sense to think in a futuristic vision, which includes the incorporation of these biosensors into portable lateral flow strip-like devices or in exploiting biochemical computing and logic-gate systems to offer “Sense/Act” operation devices for rapid analysis of target analytes directly on-site at the crime scene.

## Figures and Tables

**Table 1:** Electrochemical biosensors for the determination of arsenic and cyanide.

Electrode	Analyte/Sample	Method	Transduction Technique	Analytical Characteristics	Ref.
GA/SPCE	As(III) and As(V)/waters	Immobilization of AcChE and AcP; measurements based on the respective inhibitory effects on enzymes activity of As(III) using ATI and TTF, and As(V) using 2-phospho-l-ascorbic	Amperometry, 150 (III), 250 mV (V) vs. Ag/AgCl	LR: 0.2–1.6 mM; 35.9–352.9 μM (III); 2.0–19.6 μM; 20–160 μM (V); LOD: 28.7 μM (III); 1.2 μM (V)	[24]
AuE	As(III)/spiked water	Preparation of ssDNA/SWCNT conjugates. Dissociation in presence of As, assembling of liberated SWCNTs onto AuE and increasing conductivity	DPV, FcCOOH	LR: 5–10 μg·L^−1^ LOD: 0.5 μg·L^−1^	[25]
AuNPs/Chit/SPCE	As(III)/Waters	Immobilization of As specific aptamer and adsorption of PDDA. Measurement of the conductivity increase in the presence of As by desorption of PDDA	DPV, Ru(NH_3_)_6_^3+^	LR: 0.2–100 nM LOD: 0.15 nM	[26]
AuE	As(III)/Waters	Immobilization of ssDNA_cap_, hybridization with As specific aptamer AptH0, and with H1 and H2 strands. Measurements of decreasing R_CT_ by interaction with As and dissociation of the dsDNA_cap_. Amplification by digestion with RecJf exonuclease.	EIS, Fe(CN)_6_^3−/4−^	LR: 0.1–500 μg·L^−1^ LOD: 0.02 μg·L^−1^	[20]
3D-rGO/AuNPs/GCE	As(III)/Water	Immobilization of a thiolated aptamer and measurement of electron transfer hindrance in presence of the target. Amplification with GA and HOOC-CNTs-BSA	EIS, Fe(CN)_6_^3−/4−^	LR: 3.8 × 10 ^−^^7^–3.0 × 10^−^^4^ ng·mL^−1^ LOD: 1.4 × 10^−7^ ng·mL^−1^	[19]
GA/Nf/Chit/GCE	As(III)/Waters	Immobilization of ssDNA_cap_ and hybridization with the As specific aptamer. Measurements of ΔR_CT_ in presence of different concentrations of arsenic	EIS, Fe(CN)_6_^3−/4−^	LR: 0.15–10; 20–100 nM LOD: 74 pM	[18]
HRP/AuSNPs/SNGCE	CN^−^/-	Immobilization of HRP and measurements based on the inhibitory effect of cyanide on the enzyme activity using caffeic acid as substrate	Amperometry, –0.15 V vs. Ag/AgCl	LR: 0.1–58.6 μM LOD: 0.03 μM	[21]
GA/PANI/PtE	CN/artificial waste water	Immobilization of CAT and measurements based on the inhibitory effect of cyanide on the enzyme activity using H_2_O_2_ as substrate	EIS, Fe(CN)_6_^3−/4−^	LR: 0.0136–0.65 mg·L^−1^ LOD: 2 μg·L^−1^	[22]
NH_4_^+^-ISE	CN/industrial wastewater, food	Immobilization of *Flavobacterium indicum* whole cells. Measurement of ammonium produced by cyanide dehydratase of the cells proportional to target concentration.	Potentiometry	LR. 10^−10^–0.1 M LOD: 1 nM	[23]

**Abbreviations:** 3D-rGO: three-dimensional reduced graphene oxide; ATI: acetylthiocholine; AuNPs: gold nanoparticles; AuSNPs: gold sononanoparticles; CAT: catalase; Chit: chitosan; EIS: electrochemical impedance spectroscopy; GA: glutaraldehyde; GCE: glassy carbon electrode; HRP: horseradish peroxidase; ISE: ion selective electrode; Nf: Nafion; LOD: limit of detection; LR: linear range; PANI: polyaniline; PDDA: poly-diallyl dimethyl ammonium; PtE: platinum electrode; SNGCE: sonogel carbon electrode; SPCE: screen-printed carbon electrode; TTF: tetrathiafulvalene.

**Table 2:** Electrochemical biosensors for ethanol and ethanol metabolites.

Electrode	Analyte/Sample	Method	Transduction Technique	Analytical Characteristics	Ref.
Fe_3_O_4_@AuNPs/MnO_2_/CPE	ethanol/beverages	Immobilization of ADH and detection of NADH in the presence of NAD^+^	Amperometry, 0.1 V vs. Ag/AgCl	LR: 0.1–2.0 M LOD: 0.07M	[41]
TOA-AuNPs/Azure A-SPCE	ethanol/wine	Immobilization of ADH; covering with chitosan and voltammetric measurements in the presence of NAD^+^	DPV, NADH	LR: 0.001–2.0 mM LOD: 0.14 mM	[42]
PPy-PVS/PtE	ethanol/beverages	Immobilization of ADH and NAD^+^; NADH detection with Meldola’s blue as redox mediator	Amperometry, −0.072 V vs. Ag/AgCl	LR: 1.0–10.0 µM; 0.01–0.1 mM; LOD: 0.1 μM	[43]
PtNPs/MnOx-MoOx/GCE	ethanol/beverages	Immobilization of *Gluconobacter oxydans*. Monitoring of oxygen consumption	Amperometry, 0.0 V vs. Ag/AgCl	LR: 0.075–5.0 mM	[33]
wearable tattoo with PB carbon ink	ethanol/sweat	Sweat induction with pilocarpine and iontophoretic biosensing with AOx	Amperometry, −0.2 V vs. Ag/AgCl	LR: up to 36 mM	[38]
PNR/AuNPs/MWCNTs/SPCE	ethanol/beverages	Immobilization of ADH and detection of NADH in the presence of NAD^+^	Amperometry, 0.2 V vs. Ag/AgCl	LR: 0.32–1.0 mM LOD: 0.096 mM	[32]
polyTyr/SWCNTs/GCE	ethanol/beverages	Immobilization of ADH by entrapment with Nafion and NADH detection in the presence of NAD^+^	Amperometry, 0.2 V vs. Ag/AgCl	LR: 0.01–0.15 mM LOD: 0.67 mM	[31]
wearable Au or ZnO electrodes onto glass or polyimide	EtG/sweat	Immobilization of EtG antibody using thiol-based chemistry. Measurement of impedance changes	EIS	LR: 0.001–100 μg/L LOD: 1 μg·L^−1^ (AuE); 0.001 μg·L^−1^ (ZnO)	[35]
PDA/Fe_3_O_4_/GCE	ethanol/human serum	Immobilization of AOx; detection of H_2_O_2_ as substrate	Amperometry, −0.1 V vs. Ag/AgCl	LR: 0.5–3.0 mM LOD: 130 μM	[44]
smartphone-based platform with PtEs	ethanol/blood	Electrodeposition of HRP and AOx onto calcium alginate; H_2_O_2_ detection with TMB as redox mediator	Amperometry, 0.0 V vs. Pt	LR: up to 1.25 g L^-1^ LOD: 0.056 g L^-1^	[40]
Pt-Ru	ethanol/serum, saliva	ADH immobilized on a dialysis membrane in the anode of the fuel cell	Amperometry	LR: 0.5–600 mM LOD: 0.2 mM	[45]
ZnO	ethanol/sweat	Immobilization of AOx and measuring of impedance changes	EIS	LR: 0.01–200 mg·dL^−1^ LOD: 0.01 mg·dL^−1^	[39]
ZnO-NFs/Au/pET	EtG	Immobilization of EtG antibody via electrostatic interaction	CV, EIS [Fe(CN)_6_]^3−/4-^	LR: 1 ng·mL^−1^-100 μg·mL^−1^ LOD: <1 ng·mL^−1^	[46]

**Abbreviations:** CPE: carbon paste electrode; EtG: ethyl glucuronide; LOD: limit of detection; LR: linear range; NFs: nanoflakes; PB: Prussian blue; PDA: polydopamine; pET: polyethylene terephthalate; PNR: polyneutral red; PPy-PVS: polypyrrole-polyvinyl sulfonate; TMB: 3,3′,5,5′-tetramethylbenzidine; TOA: thioctic acid.

**Table 3:** Electrochemical biosensors for the determination of toxins.

Electrode	Analyte/Sample	Method	Transduction Technique	Analytical Characteristics	Ref.
SPCE	AFM1/milk	Label-free aptasensor. Apt immobilization by diazonium-coupling. R_CT_ measurements in the presence of AFM1	EIS, [Fe(CN)_6_]^3−/4−^	LR: 2–150 ng·L^−1^ LOD: 1.15 ng·L^−1^	[93]
SPAuE	AFM1/milk, serum	Apt immobilization onto SPAuE; Apt CS conjugation with AuNPs. Disassembled of Apt hairpin structure in presence of AFM1 and current increasing with MB as redox agent	DPV, MB	LR: 2–600 pg·mL^−1^ LOD: 0.9 pg·mL^−1^	[80]
Chit/AuNP/disk-ring AuμE	AFB1/wheat	Label-free immunosensor. Immobilization of anti-AFB1 and current measurement after conjugation with the antigen	CV, [Fe(CN)_6_]^3−/4−^	LR: 0.2–2, 2–30 ng·mL^−1^ LOD: 0.12 ng·mL^−1^	[94]
PDMS/SPCE	AFB1/peanuts	Immobilization of thiolated Apt onto Fe_3_O_4_@Au and assembling on PDMS/SPCE. Measurement of impedance changes	EIS, [Fe(CN)_6_]^3−/4−^	LR: 20–5 × 10^4^ pg·mL^−1^ LOD: 15 pg·mL^−1^	[79]
SPCE	OTA/cocoa beans	Label-free aptasensor. Apt immobilization by diazonium-coupling. R_CT_ measurements in the presence of OTA	EIS, [Fe(CN)_6_]^3−/4−^	LR: 0.15–2.5 ng·mL^−1^ LOD: 0.15 ng·mL^−1^	[95]
Cyst-GCE	OTA/soybean	Immobilization of cDNA onto AuNPs-Cyst-cPC and drop onto Cyst-GCE to hybridize with the Apt. R_CT_ measurements in the presence of OTA	EIS, [Fe(CN)_6_]^3−/4−^	LR: 10^−8^–0.1 ng·mL^−1^ LOD: 10^−8^ ng·mL^−1^	[96]
SPCE	OTA/coffee	Grafting of PT3C or PP3C onto SPCE and covalent immobilization of Apt to complex OTA increasing R_CT_	EIS, [Fe(CN)_6_]^3−/4−^	LR: 0.125–2.5 ng·mL^−1^ LOD: 0.125 ng·mL^−1^	[97]
OctAuNPs/GCE	OTA/wine	Immobilization of Ab_1_ onto OctAuNPs/GCE. OTA sandwiched with AuOct PCs-TB@Ab_2_ as carrier tag for signal amplification	SWV, TB	LR: 0.1–10^4^ pg·mL^−1^ LOD: 39 fg·mL^−1^	[84]
AuE	OTA/wine	DNA-controlled layer-by-layer assembly of dual AuNPs conjugates using capture probes to hybridize Apt and Fc tagged SH-signal probe	DPV, Fc	LR: 0.001–500 ng·mL^−1^ LOD: 0.001 ng·mL^−1^	[98]
*β*-CD-SH-SPAuE	OTA/wine	Apt hybridization with cDNA-MB. Apt-OTA complexation, cDNA-MB separation. Target recycling signal amplification by RecJf exonuclease	DPV, MB	LR: 10–10^4^ pg·mL^−1^ LOD: 3 pg·mL^−1^	[99]
Fe_2_O_3_/MCM-41/SPCE	ZEA/seeds	Sandwich-type immunoassay. Immobilization of anti-ZEA onto Fe_2_O_3_/MCM-41/SPCE and conjugation with HRP-anti-ZEA. Current measurements by addition of H_2_O_2_/4-TBC	Amperometry, −0.1 V vs. Ag/AgCl	LR: 1.88–45 ng·mL^−1^ LOD: 0.57 ng·mL^−1^	[100]
AuE	ZEA/–	Flow-injection capacitive immunosensor. Immobilization of anti-ZEN onto pTYR or 3-MPA or LA SAMs-modified AuE	Capacitance current-pulse FI	LR: 0.01–10 nM (pTYR); 0.02–10 nM (SAMs) LOD: 0.006 nM (LA SAM)	[101]
Chit/SWCNT/GCE	DON/sorghum, infant food	Indirect competitive immunosensor. Detection with AP-IgG, using 1-NPP as substrate	DPV, 1-NP	LR: 0.01–1000 ng·mL^−1^ LOD: 5 pg·mL^−1^	[102]
AuNPs/PPy/ErGO/SPCE	FB1 and DON	Label-free immunosensor. Immobilization of antitoxin onto the modified electrode and R_CT_ measurements	DPV, [Fe(CN)_6_]^3−/4−^	LR: 0.2–4.5 (FB1), 0.05–1 ng·mL^−1^ (DON); LOD: 4.2 (FB1) 8.6 ng·L^−1^ (DON)	[103]
PoAP/CNT/SPCE	OA/shellfish	Enzyme biosensor based on inhibition of PP2A and voltammetric detection after addition of 1-NPP	DPV, 1-NP	LR: 1–300 μg·L^−1^ LOD: 0.55 μg·L^−1^	[89]
Phosphorene-gold/SPCE	OA/mussel	Microfluidic biochip of OA. Immobilization of Apt. Current decreasing in presence of OA	DPV, [Fe(CN)_6_]^3−/4−^	LR: 10–250 nM LOD: 8 nM	[104]
PDIC/Cyst/AuE	BTX-2	Aptasensor. Immobilization of BTX-2 and competitive assay between BTX-2 onto electrode and free BTX-2 in presence of a fixed amount of Apt	EIS, [Fe(CN)_6_]^3−/4−^	LR: 0.1–100 ng·L^−1^ LOD: 106 pg·mL^−1^	[105]
MB-cMWCNTs/ODT/AuE	STX/mussel	Label-free aptasensor. Target-induced conformational change of Apt with STX binding. Measurement of current decreasing in presence of toxin	DPV/ MB	LR: 0.9–30 nM LOD: 0.38 nM	[106]
lipid film/ graphene	STX/lake water, shellfish	Potentiometric immunosensor. Immobilization of anti-STX onto a lipid film prepared by polymerization in a mixture of DPPC, MA, EGDM and AMPN	Potentiometry, stopped-flow	LR: 1.3 × 10^−9^–1.3 × 10^−6^ M LOD: 1 nM	[87]
MGE	STX/seawater, shellfish	Sandwich-type magnetoimmunosensor. Biotin-Ab_2_ immobilization onto Avidin-MBs. Conjugation with Ab_1_, STX complexation and interaction with (g-C_3_N_4_-PdNPs). Current measurements by addition of H_2_O_2_/TMB	Amperometry, 0.2 V vs. Ag/AgCl	LOD: 1.2 pg·mL^−1^	[107]
HOOC-PEG6-DTA/SPAuEa	TTX/putter fish	TTX immobilization onto activated carboxylate-dithiol. Addition of cAb and IgG-HRP. Current measurements in presence of TMB	Amperometry, −0.11 V vs. Ag	LR: 2.6–10.2 ng·mL^−1^ LOD: 2.6 ng·mL^−1^	[86]
SPCEa	TTX/putter fish	TTX immobilization on Cyst-maleimide-MBs. Addition of cAb and IgG-HRP. Current measurements in presence of TMB	Amperometry, −0.2 V vs. Ag	LR: 1.2–52.7 ng·mL^−1^ LOD: 1.2 ng·mL^−1^	[108]
cSWCNTs/ Chit/AuNPs/ GCE	T-2 toxin/feed, swine meat	Immunosensor. Competitive assay between T-2 and OVA-T-2-cSWCNTs. Detection by AP-Ab_2_ and 1-NPP	DPV, 1-NP	LR: 0.01–100 μg·L^−1^ LOD: 0.13 μg·mL^−1^	[109]
pDA/AuNRs magnetic rGO	MC-LR/water	Competitive immunosensor. Immobilization of antibody and rolling circle DNA amplification	DPV; H_2_O_2_/HQ	LR: 0.01–50 μg·L^−1^ LOD: 0.007 μg·mL^−1^	[110]
AuNDs/ITO	MC-LR/−	Label-free immunosensor. Conjugation of Ab and sDNA to (SiO_2_@MSN). HCR to form G-quadruplex/hemin. MB intercalation.	DPV, H_2_O_2_	LR: 0.5 ng·L^−1^–25 μg·L^−1^ LOD: 0.3 ng·L^−1^	[92]
PET/graphene/Cu	MC-LR/waters	Label-free immunosensor involving covalent immobilization of MC-LR onto oxidized electrode and competitive assay between immobilized and free antigen in presence of a fixed amount of antibody	EIS, [Fe(CN)_6_]^3−/4−^	LR: 0.005–10 μg·L^−1^ LOD: 2.3 ng·mL^−1^	[111]
AuE	MC-LR/water	Label-free DNA biosensor. Immobilization of calf thymus DNA and measurement of R_CT_ decrease in presence of MC-LR	EIS, [Fe(CN)_6_]^3−/4−^	LR: 4.0–512 ng·L^−1^ LOD: 1.4 ng·L^−1^	[112]
Cyst/AuE	MC-LR/cyano-bacteria culture	Microfluidic immunosensor. Immobilization of MC-LR. Competitive assay between immobilized and free antigen with a fixed amount of antibody	EIS, [Fe(CN)_6_]^3−/4−^	LR: 0.1–330 μg·L^−1^ LOD: 0.57 ng·L^−1^	[113]

**Abbreviations:** AFM1: aflatoxin M1; AMPN: 2,2′-azobis-(2-methylpropionitrile); AP: alkaline phosphatase; Apt: aptamer; AuNDs: gold nanodendrites; AuNRs: gold nanorods; AuOct PCs: gold octahedron plasmonic colloidosomes; *β*-CD: beta-cyclodextrin; Chit: chitosan; cMWCNTs: carboxylated multiwalled carbon nanotubes; CS: complementary strand; cSWCNTs: carboxylated single-walled carbon nanotubes; DON: deoxyvalenol; DPPC: dipalmitoyl phosphatidylcholine; DPV: differential pulse voltammetry; EGDM: ethylene glycol dimethacrylate; Fc: ferrocene; GCE: glassy carbon electrode; HCR: hybridization chain reaction; HQ: hydroquinone; ITO: indium tin oxide electrode; LA: lipoic acid; LOD: limit of detection; LR: linear range; MA: methylacrylic acid; MB: methylene blue; MC-LR: microcystin-LR; MCM-41: amino mesoporous silica; MGE: magnetic gold electrode; 3-MPA: 3-mercaptopropionic acid; 1-NP: 1-naphthylphenol; 1-NPP: 1-naphthylphosphate; OA: okadaic acid; OctAuNPs: octahedral gold nanoparticles; ODT: octadecanethiol; OVA: ovalbumin; pDA:polydopamine; PDIC: 4-phenylene diisocyanate; PDMS: polydimethylsiloxane; DTA: dithioalkane aromatic; PET: polyethylene terephthalate; PoAP: poly-o-aminophenol; PP2A: protein phosphatase 2A; pTYR: polytyramine; R_CT_: charge transfer resistance; rGO: reduced graphene oxide; SPAuEa: screen-printed gold electrode array; SPCEa: screen-printed carbon electrode array; STX: saxitoxin; TB: toluidine blue; 4-TBC: 4-terbutylcatechol; ZEA: zearalenone.
